# Calcitriol Modulates Hippocampal Axon Guidance Through Enhanced EfnA4‐Mediated PI3K/AKT Signaling in an Autism Mouse Model

**DOI:** 10.1111/cns.70429

**Published:** 2025-05-21

**Authors:** Tiantian Gong, Ruizhen Sun, Jieli Bai, Xin Liu, Chenyao He, Qi Jiang, Qi Wang, Yubo Qi, Wenxin Ding, Jingling Shen, Lei Lei, Zhiyan Shan

**Affiliations:** ^1^ Department of Histology and Embryology, School of Basic Medical Sciences Harbin Medical University Harbin China; ^2^ Guangzhou Laboratory Guangzhou International Bio Island Guangzhou China; ^3^ Institute of Life Sciences, College of Life and Environmental Science Wenzhou University Wenzhou China

**Keywords:** autism spectrum disorder, axon guidance, behaviors, calcitriol, hippocampus

## Abstract

**Aims:**

Autism spectrum disorder (ASD) is a complex neurodevelopmental condition arising from the interplay of genetic predispositions and environmental influences. Recent studies have suggested that vitamin D (VitD) supplementation play a role in reducing the risk of ASD and alleviating some of its core symptoms. However, variations in individual responses to VitD due to biological heterogeneity have led to inconsistent clinical outcomes, and the precise molecular mechanisms through which VitD might exert its effects on ASD remain poorly understood.

**Methods:**

We investigated the effects of calcitriol, the biologically active form of VitD, on ASD‐associated phenotypes in BTBR mice, a well‐established autism model. Behavioral assessments were used to evaluate social and repetitive behaviors. Mechanistic insights were obtained through RNA sequencing, immunohistochemistry, biochemical assays, and stripe guidance assays.

**Results:**

Calcitriol supplementation significantly improved autism‐like behaviors in BTBR mice, alleviating hippocampal hypoplasia and correcting axon guidance abnormalities. These effects were mediated by modulation of the EfnA4‐PI3K signaling pathway in hippocampal neural progenitor cells and other brain regions, highlighting its role in neurodevelopmental processes.

**Conclusion:**

Our findings demonstrate that calcitriol targets axon‐guidance‐related signaling pathways, providing a theoretical framework and potential clinical strategy for targeted ASD interventions.

## Graphical abstract

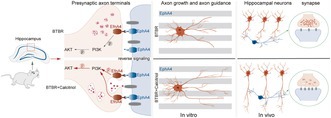

## 1 Introduction

Autism spectrum disorder (ASD), also known as autism, is a heterogeneous, behaviorally defined lifelong neurodevelopmental disorder [1]. Manifestations of autism include impairments in social communication and interaction, repetitive behaviors, and varying levels of intellectual disability [2, 3]. Despite a significant increase in opinion and diagnosis of ASD over recent decades, its underlying pathophysiology remains largely elusive [4]. Currently, there are no specifically targeted pharmacological treatments available for this disorder [5], highlighting an urgent need for novel therapeutic approaches. This limitation has driven extensive research focused on identifying modifiable risk factors and biological pathways that may contribute to the onset and progression of ASD [6]. Among the factors under investigation, vitamin D (VitD) deficiency has emerged as a compelling candidate [7], implicated not only as a potential risk factor for ASD but also as a therapeutic target for alleviating its symptoms [8, 9].

VitD is primarily obtained through dietary intake and sunlight exposure, undergoing hydroxylation in the liver and kidneys to produce its biologically active form 1,25‐dihydroxyvitamin D3 (calcitriol, D3) [10]. Calcitriol exerts its biological effects on target cells through both genomic pathways mediated by binding to the nuclear vitamin D receptors (VDR) and non‐genomic pathways mediated by membrane‐associated VDR, influencing a diverse array of cellular functions critical for brain health and neural development [11]. Observational studies have correlated VitD deficiency with increased severity of ASD symptoms, while VitD supplementation in children with ASD has shown promise in improving ABC (autism behavior checklist) and CARS (childhood autism rating scale) [12, 13]. Furthermore, animal studies suggest a preventive potential for early VitD supplementation, with maternal VitD intake linked to reduced ASD phenotypes in offspring [14]. Despite these findings, the precise mechanisms through which VitD may exert neuroprotective effects in ASD remain imperfectly understood, retarding the development of targeted VitD‐based interventions.

Recently, research on neurodevelopmental guidance molecules has elucidated additional pathways that may be implicated in ASD [15]. The Ephrin family of guidance cues and their corresponding Eph receptors play crucial roles in regulating key aspects of neurodevelopment, including axonal and dendritic growth, synaptic formation, and neural circuit assembly [16]. Dysregulation of Ephrin–Eph interaction signaling has been associated with ASD and other neurodevelopmental disorders, exhibiting significant behavioral parallels to core ASD symptoms [17]. For instance, loss of Ephrin‐A2 in murine models results in impaired behavioral flexibility, a hallmark of ASD, while combined deletions of Ephrin‐A2 and ‐A3 yield repetitive behaviors closely resembling those observed in the disorder [18, 19]. Notably, Ephrin‐A's functional plasticity in neuronal growth processes allows it to exhibit either attractant or repellent effects depending on its concentration and the cellular microenvironment, indicating its dynamic role in axonal outgrowth. Although advances in understanding Ephrin signaling in ASD, critical gaps remain regarding the specific Ephrin‐A subtypes and their downstream molecular pathways involved in the disorder [20].

In this study, we aim to address these gaps by investigating the effects of calcitriol on ASD‐like behaviors and neurodevelopmental abnormalities in the BTBR mouse model, a widely validated model of ASD. We hypothesize that calcitriol may ameliorate ASD‐related behavioral and neurodevelopmental deficits through modulation of the Ephrin‐A‐Eph signaling pathway. Our results demonstrated that calcitriol administration significantly improved core ASD‐like behaviors, including social deficits and stereotypic behavior, in BTBR mice. Mechanistically, we observed that BTBR neurons exhibit decreased expression of EfnA4 (Ephrin‐A4) and reduced responsiveness to EphA4, collectively disrupting axonal guidance and branching within the hippocampus, a brain region essential for cognitive and social behavior. Importantly, calcitriol treatment reversed these deficits by activating the EfnA4‐PI3K‐AKT signaling cascade, leading to improved neurite growth and connectivity in the hippocampus. Collectively, our findings provide novel insights into the neuroprotective potential of calcitriol in ASD, revealing a previously unidentified pathway—EfnA4‐PI3K‐AKT signaling—that may underlie the therapeutic benefits of VitD in neurodevelopmental disorders.

## 2 Methods and Materials

### 2.1 Animals

The BTBR T^+^Itpr3^tf^/J (BTBR) and C57BL/6J (C57) mice were purchased from Jackson Laboratory in the United States and Beijing Vital River Laboratory Animal Technology Co. Ltd., respectively. The laboratory maintained an ambient temperature of 21°C–23°C and a relative humidity of 50%–70%. They were provided with sufficient food and water while being raised on a 12h –12 h light–dark cycle. Only male mice that were 5 weeks old and weighed between 18g and 25 g were used for this study. All animals were assigned randomly to various experimental groups.

### 2.2 Drug Treatment

Calcitriol (Sigma‐Aldrich) was dissolved in 5% dimethyl sulfoxide and 0.9% normal saline and given to mice intraperitoneally at different doses (0.5 μg/kg, 1.5 μg/kg, 3 μg/kg). The male BTBR mice were divided randomly into four groups at the age of 5 weeks and were injected with either calcitriol or saline solution every other day for 6 consecutive days. Then, the mice were subjected to behavioral tests at D6 and D12. Simultaneously, age‐matched male C57 mice served as the normal control group, and each group consisted of 15–20 mice.

### 2.3 Intracranial Stereotaxic Injections of Lentivirus

Bilateral BTBR hippocampi received injections of lentivirus (anteroposterior (AP): −2.2 mm; mediolateral (ML): ±1.5 mm; dorsoventral (DV): 2.2 mm) delivered in a volume of 1.2 μL at a flow rate of 0.2 μL/min. One week after stereotaxic surgery, the mice were subjected to behavioral assessments.

### 2.4 Behavioral Tests

Behavioral assessments including the three‐chamber social test, grooming test, and marble burying test were conducted before and after injection. Transfer the mice to the procedure room (for acclimation) 1 h before testing. The apparatus was carefully cleaned by wiping it with 75% ethanol after each experiment to remove the olfactory distraction. All mice were familiarized with the researchers at least 3 days in advance. Detailed descriptions can be found in the Supporting Information.

**FIGURE 1:**
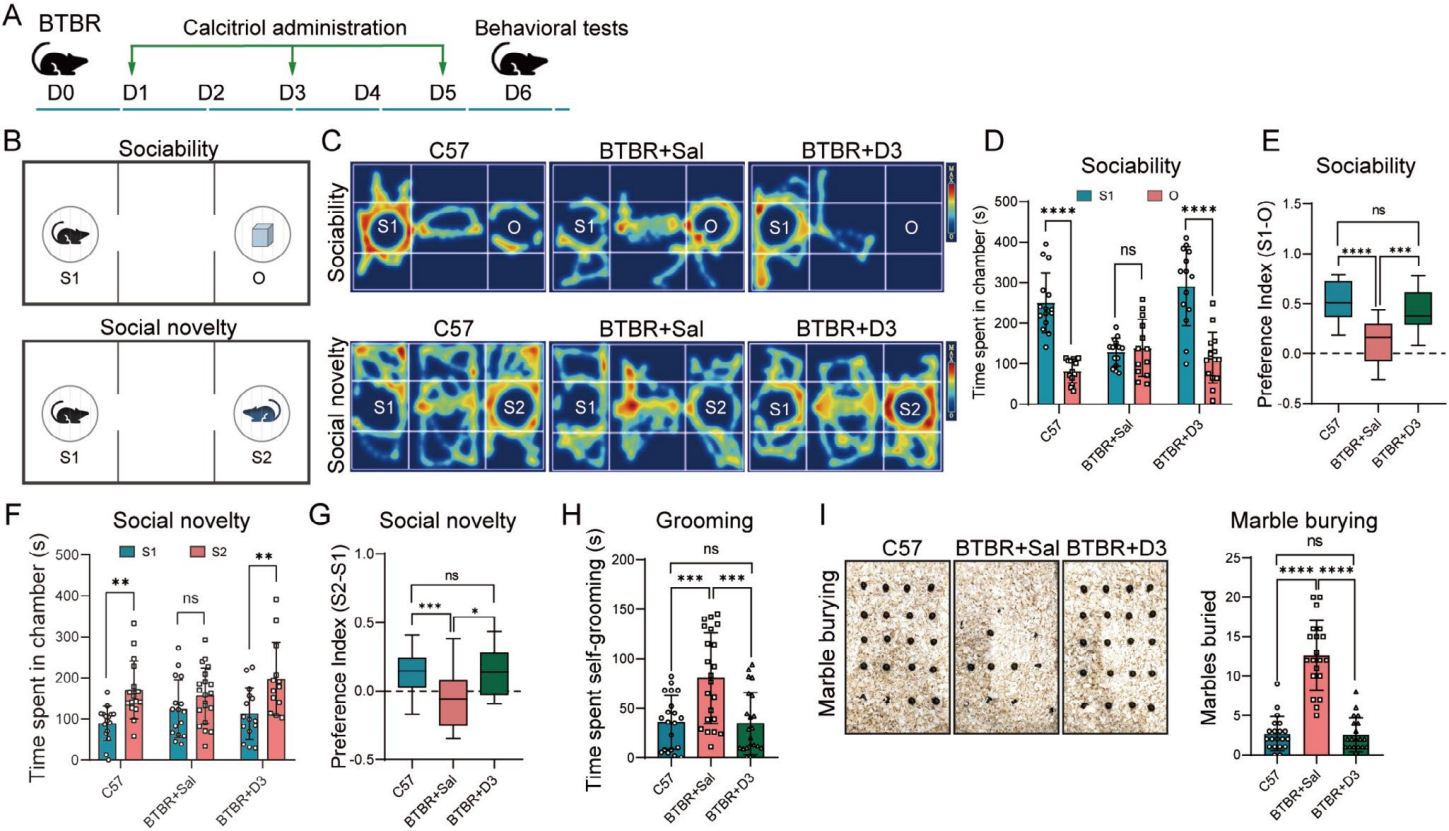
Calcitriol alleviates autistic‐like behaviors in BTBR mice. (A) Schematic representation illustrating the experiment design. (B) Pattern diagram for the sociability and social novelty testing stage. (C) Representative tracing heatmap analysis of C57 mice and BTBR mice treated with saline (BTBR + Sal) or calcitriol (BTBR + D3) during the three‐chamber social novelty test. (D) Total interaction time with familiar mouse (S1) or object (O) during the three‐chamber test. BTBR + D3 mice showed increased sociability compared with BTBR + Sal mice. (E) Social preference index in the stage of sociability. (F) Using the three‐chamber paradigm, the social novelty test was performed in the three groups. BTBR + D3 mice spent more time with the novel mouse (S2) than with the familiar mouse (S1). (G) Social preference index in the stage of social novelty. (H) Grooming test for the three groups. (I) Representative results in the marble burying test (left). BTBR + D3 mice showed decreased numbers of marbles buried (right). Statistical significance is denoted as ns (no significance), *n* > 10 in each group, *p < 0.05, **p < 0.01, ***p < 0.001, ****p < 0.0001. Data are presented as mean ± SEM.

### 2.5 Immunofluorescence

After deparaffinization and rehydration, the brain slides were subjected to heat‐mediated antigen retrieval in a 10 mM sodium‐citrate solution (Proteintech) at 95°C for 10 min using a microwave oven. Then, slides were incubated for 1 h in a blocking solution, and the primary antibody was applied by diluting it to sections and incubated overnight at 4°C. The next day, sections were applied with corresponding secondary antibodies for 30 min and were coverslipped with Hoechst33342 (Beyotime). The information and dilution of antibodies was available in Table S1.

### 2.6 Transmission Electron Microscopy

The hippocampus was dissected and fixed with 2.5% buffered glutaraldehyde followed by 1% osmium tetroxide. The samples were dehydrated in ascending concentrations of ethanol and embedded in Epon 812. The synapse structures were observed under a transmission electron microscope. Photos of five sections were obtained for morphometric analysis using Image‐Pro Plus 6.0 software. Synapse features, including post synaptic density (PSD), synaptic cleft width, and presynaptic active zone, were measured.

### 2.7 RNA Seq Analysis

RNA was extracted from the mouse hippocampus using Trizol Reagent (Thermo Fisher Scientific). After assessing the RNA quality, a sequencing library was created and sequenced on the NovaSeq 6000 platform (Illumina) by Annoroad Gene Technology Co. Ltd. Using the edgeR software, we analyzed the differentially expressed genes (DEGs) between the three groups of C57, BTBR, and calcitriol by a *p*‐value cutoff of 0.05 and a fold‐change cutoff of 1.5 for statistical analysis.

### 2.8 Quantitative Real‐Time PCR (qPCR)

Total RNA was harvested using Trizol Reagent (Thermo Fisher Scientific), and reverse transcription was performed using One‐Step gDNA Removal (TransGen). The SYBR Green kit (TransGen) was used for qPCR. All of the samples were diluted to 1:3 with RNase/DNase‐free water, and 3 μL were used for each SYBR Green qPCR reaction that also contained 10 μL 2 × *TransStart* Top Green qPCR SuperMix, 10 μM of each primer, and deionized water. Primers are listed in Table S2.

### 2.9 Western Blot

Mouse hippocampal tissue was extracted by the lysis buffer, with 200 μL of the lysis working solution added for every 10 mg of tissue. The supernatant was collected after centrifugation at 12,000 rpm for 30 min. Protein samples were electrophoresed and transferred onto a PVDF membrane. The membranes were blocked with 5% skimmed milk for 1 h at RT, incubated overnight at 4°C with the primary antibodies, and then incubated with corresponding secondary antibodies for 1 h at RT. Aimed protein bands in the membranes were visualized through the chemiluminescence method (Thermo Fisher Scientific) and quantified using ImageJ software. Additional antibody information is available in Table S1.

### 2.10 Statistical Analysis

Statistical analysis was conducted using GraphPad Prism software. Data were presented as the mean ± standard error of the mean (SEM). The Shapiro–Wilk test was used to assess the normality of the data distribution. Parametric tests including t‐tests and one‐way analysis of variance (ANOVA) were used if the data were normally distributed, and nonparametric approaches, including the Mann–Whitney test and Kruskal–Wallis test, were used for data with a nonnormal distribution. Statistical significance was defined as *p* < 0.05.

## 3 Results

### 3.1 Calcitriol Treatment Alleviates Autism‐Like Behaviors in BTBR Mice

BTBR mice, especially male individuals, display cognitive and social impairments similar to those found in ASD patients, allowing for a precise evaluation of the selected treatment impact on ASD‐linked behavioral symptoms. To examine whether a treatment with calcitriol corrected the behavioral impairments, adult male BTBR mice were injected with either calcitriol or saline on desired days and later tested in a battery of behavioral assays. Since deficits in social interaction as well as repetitive and restricted behaviors are two major clinical manifestations found in human ASD patients, we first focused on testing these core autism‐like behaviors. We used the three‐chamber assay to examine the sociability and social novelty of calcitriol‐and saline‐treated mice (Figure 1A–C). Planned comparisons revealed that the BTBR mice did not show a preference for the social stimulus; in contrast, the 1.5 μg/kg calcitriol group spent more time with the social stimulus than with the object, representing improved sociability (Figure 1D,E). In the social novelty portion of the test, the BTBR mice showed no preference, while the 1.5 μg/kg calcitriol‐treated mice preferred a novel over a familiar animal, which was similar to normalities observed in C57 mice (Figure 1F,G). In addition, we observed that all 1.5 μg/kg calcitriol‐treated mice displayed decreased grooming and marble burying episodes approaching C57 mice, indicating a reduction in stereotyped and repetitive behaviors (Figure 1H,I). It should also be noted that the 0.5 μg/kg calcitriol treatment did not show significant therapeutic effects. Although a dose of 3 μg/kg calcitriol could effectively improve the social behavior of BTBR mice, it led to a sharp decline in their body weight and even caused the death of some mice (Figure S1A–D). Together, the collective observations and behavior tests revealed that the 1.5 μg/kg treatment with calcitriol rescued autistic‐like behaviors, which were characterized by increasing sociability and interest in social novelty, as well as repetitive and compulsive behaviors.

### 3.2 Calcitriol Improves Hippocampal Morphology and Synaptic Activity

**FIGURE 2:**
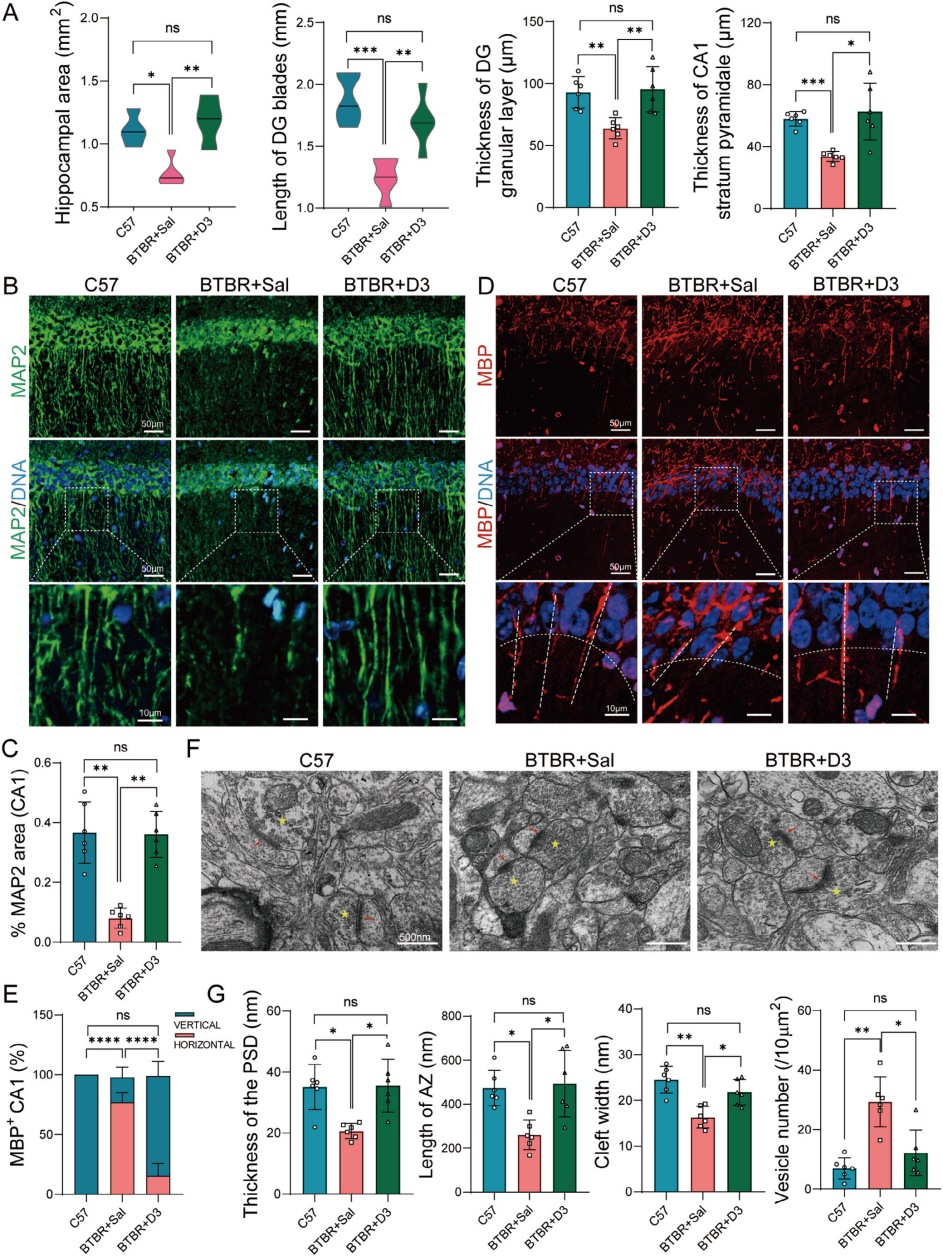
Calcitriol modulates neurite growth orientation and synaptic activity in BTBR hippocampus. (A) Quantification of hippocampal area, the length of dentate gyrus (DG) and the thickness of DG granular layer and CA1 stratum pyramidale in C57 and BTBR mice treated with saline (BTBR + Sal) or calcitriol (BTBR + D3). *n* = 6. (B) Representative images showing MAP2^+^ neurons in CA1 region of the three groups. Bottom panels: Higher‐magnification images of the areas indicated by white boxes. (C) Quantification of MAP2^+^ neurons in the three groups reveals a significant reduction in BTBR mice, which was reversed by calcitriol treatment. *n* = 6. (D) Representative images of MBP^+^ neurons (red) and quantification of axonal orientation in CA1 region in the three groups. Bottom panels: Higher‐magnification images of the areas indicated by white boxes. (E) Statistical analysis reveals a significant reduction of vertical axon in BTBR mice, which was reversed by calcitriol treatment. The axonal orientation was considered as horizontal when the angle parallel to stratum pyramidale was ≤ 45°, whereas it was considered to be vertical when angles ranged between 45° and 90°. *n* = 6. (F) Electron micrographs of synapse (red arrows) in hippocampus of the three groups (yellow pentagram bars indicate vesicle). (G) Quantification of the thickness of PSD, the length of synaptic active zone (AZ), cleft width and vesicle numbers in the three groups. *n* = 6. Statistical significance is denoted as ns (no significance), *p < 0.05, **p < 0.01, ***p < 0.001, ****p < 0.0001. Data are presented as mean ± SEM.

Hippocampus, as an important region for the development of social skills, has observed substantial morphological alterations in ASD patients [21]. Here, we found BTBR mice showed a reduced hippocampal size, abnormal commissure, and corpus callosum, which are similar to those reported in previous publications [6]. Nissl‐stained sections showed both upper and lower blades of the BTBR dentate gyrus (DG) were much shorter than those in C57, with the width of the granule cell layer of the DG and the stratum pyramidalis of CA1 significantly decreased (Figure 2A, Figure S2A–C). Immunostaining revealed irregularities and ectopic dispersion of MAP2^+^ and MBP^+^ cells in the BTBR hippocampus. While the density of neurites (MAP2^+^) was decreased in BTBR mice, calcitriol treatment led to an increase in neurite growth (Figure 2B–E). In C57 mice, axons exhibited well‐organized, parallel growth with vertical orientation, whereas BTBR axons often failed to grow in an organized, parallel way upon leaving the main pyramidal layer; their distribution in the hippocampus seemed impaired. Strikingly, calcitriol‐treated brains were greatly improved in these hippocampal hypoplasia.

Moreover, we examined the synapse structure and vesicle numbers using transmission electron microscopy (TEM). BTBR hippocampus demonstrated a reduced length of the synaptic active zone, decreased thickness of the postsynaptic membrane, and a narrowed synaptic cleft, while numerous synaptic vesicles were filled in the presynaptic membrane, indicating impaired synaptic transmission function. In contrast, the synaptic structures were restored in calcitriol‐treated hippocampi (Figure 2F,G). Collectively, these findings indicate that calcitriol plays an essential role in the maintenance of axon guidance and synaptic activity.

### 3.3 Calcitriol Mediates EfnA4 and the PI3K‐AKT Pathway: A Transcriptome Analysis of Hippocampus

**FIGURE 3:**
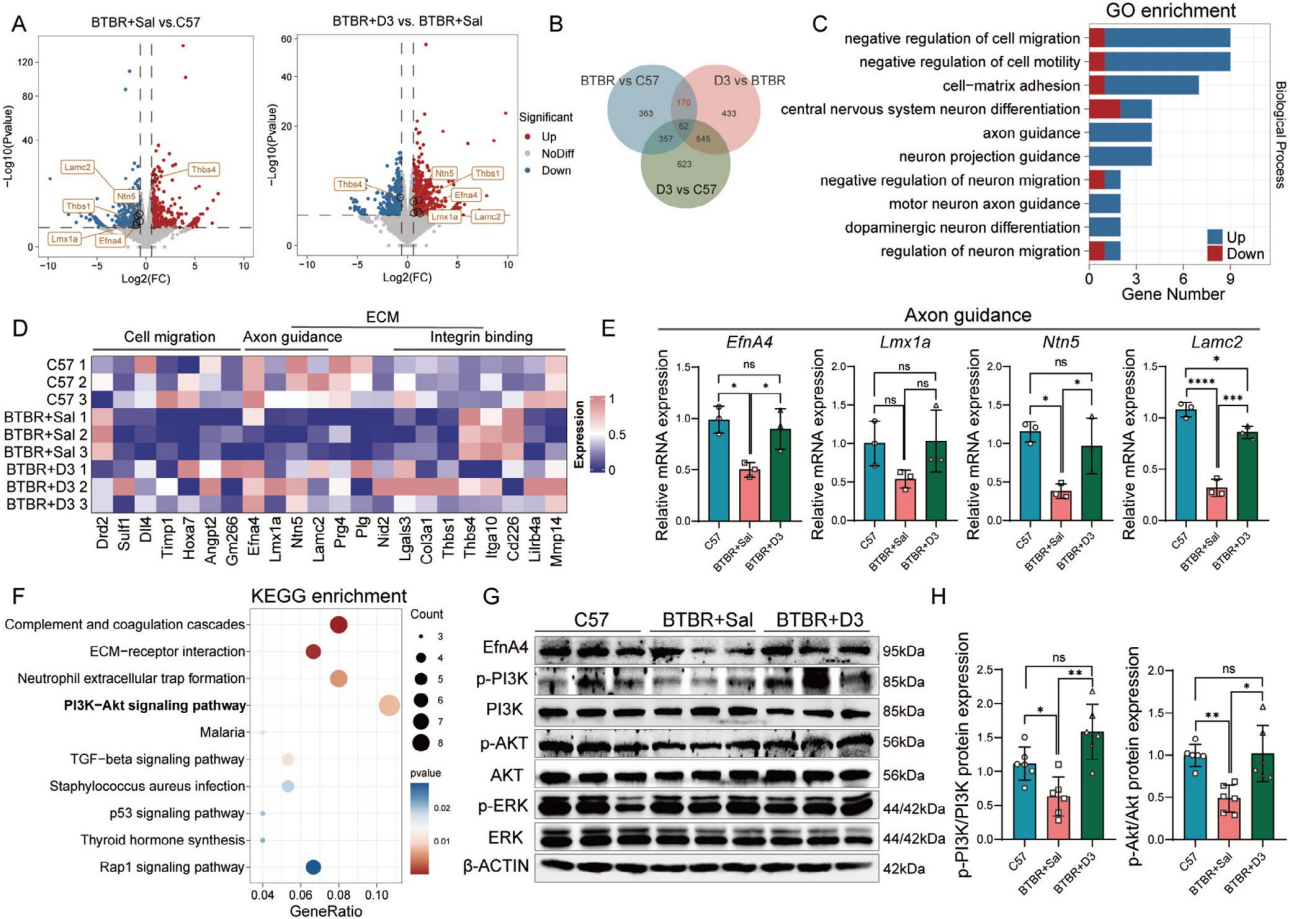
Calcitriol regulates expression of EfnA4 in BTBR mice. (A) Volcano plot representing differentially expressed genes in RNA‐seq analysis of hippocampus among BTBR + Sal mice and C57 mice (left), BTBR + D3 mice and BTBR + Sal mice (right). Red, upregulated genes; blue, downregulated genes. (B) Venn diagram showing the intersection of differentially expressed genes among the three groups, 170 genes identified as being normalized by calcitriol. (C) Gene ontology (GO) analysis for terms indicating the biological process (top 10 most significant) enriched in 170 differentially expressed genes. Blue, up‐regulated genes in BTBR + D3 group campared to BTBR + Sal group; red, down‐regulated genes in BTBR + D3 group campared to BTBR + Sal group. (D) A heatmap of gene enrichment related to functions of cell migration, axon guidance, extracellular matrix, and integrin binding within the 170 differentially expressed genes. (E) The mRNA expressions of axon guidance related genes in hippocampus among C57, BTBR + Sal and BTBR + D3. (F) The top 10 of KEGG pathway enriched in 170 differentially expressed genes. *n* = 3. (G) Western blots show for p‐PI3K, PI3K, p‐AKT, AKT, p‐ERK and ERK on hippocampus lysates from the three groups. (H) Quantification of p‐PI3K, PI3K, p‐AKT, AKT expression levels. β‐Actin was used as a loading control. *n* = 6. Statistical significance is denoted as ns (no significance), *p < 0.05, **p < 0.01, ***p < 0.001, ****p < 0.0001. Data are presented as mean ± SEM.

To address the molecular mechanisms by which calcitriol ameliorates deficits, we performed transcriptome analysis of the hippocampus by using RNA sequencing (RNA‐seq). The results identified 952 differentially expressed genes in the BTBR hippocampus compared to C57 and 1,210 differentially expressed genes in calcitriol‐treated BTBR hippocampus compared to BTBR, indicating a significant response to calcitriol supplementation (Figure 3A). We identified 170 genes from the dysregulated gene list in the BTBR hippocampus that could be rescued by calcitriol (Figure 3B). In the group of 414 genes upregulated in BTBR mice compared with C57 mice, 68 genes were found to have decreased expression in the calcitriol‐treated group and equal to that of C57(Figure S3A). Gene Ontology (GO) enrichment analysis showed that they were involved in cell migration, central nervous system neuron differentiation, and neuron migration (Figure 3C, Figure S3C). In contrast, among the 538 genes downregulated in BTBR mice compared with C57 mice, 102 genes were found to have significantly increased expression in the calcitriol‐treated group and equal to that of C57(Figure S3B), which had enrichment in GO terms related to axon guidance and neuron projection guidance (Figure 3C, Figure S3C). Further validation of key genes associated with the negative regulation of cell migration, axon guidance, and collagen‐containing extracellular matrix using qPCR supported these findings (Figure 3D,E; Figure, S3D). Results indicated that calcitriol promotes the expression of genes involved in axon growth and guidance.

Additionally, we conducted GO and KEGG enrichment analyses, the most significant enrichment was observed in the PI3K‐AKT signaling pathway (Figure 3F). Our validation confirmed that calcitriol can significantly activate the PI3K‐AKT pathway. Notably, the axon guidance‐related gene EfnA4 was significantly upregulated following calcitriol treatment and was enriched within the PI3K‐AKT signaling pathway (Figure 3G,H, Figure S3E,F). Thus, we propose that EfnA4, as an axon guidance factor, serves as a key node through which calcitriol improves ASD pathology and activates the PI3K‐AKT signaling pathway.

### 3.4 Calcitriol Improves Neurite Growth and Migration via EfnA4‐Mediated Activation of the PI3K‐AKT Pathway

**FIGURE 4:**
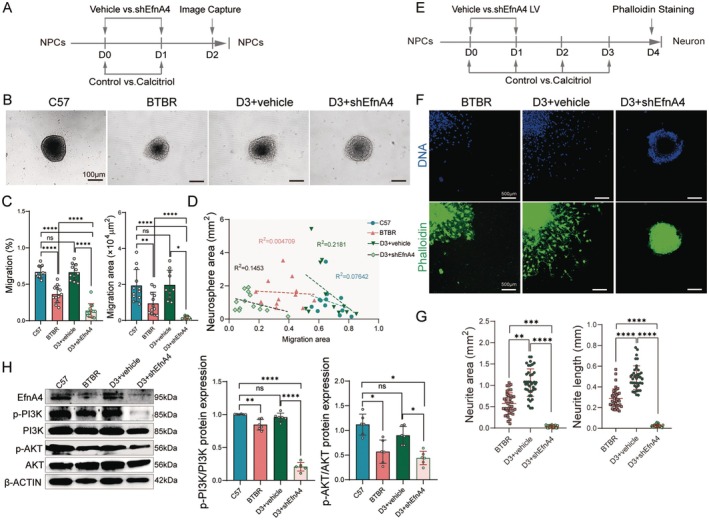
Calcitriol improves neurites outgrowth and migration through EfnA4 driven activation of PI3K‐AKT pathway in vitro. (A) Timeline of the experimental procedure. (B) Representative image showing neurospheres migration. Calcitriol treated neurospheres have migrating of cells move further than that of BTBR neurosphere, and knocking down EfnA4 (shEfnA4) reduces cell migration. (C) Quantification of NPC migration rate and migration area in C57, BTBR, D3 + vehicle and D3 + shEfnA4 group. D3 + shEfnA4 NPCs have reduced migration compared to D3 + vehicle group. *n* = 12. (D) Graph depicting the actual relationship between initial sphere size (ISS) and neurospheres migration for neurospheres (Migration~ISS, separate linear regression model for each group). *R* ^2^ values are very low indicating minimal relationship between ISS and migration. *n* = 12. (E) Timeline of the experimental procedure. (F) Fluorescent images of neurospheres differentiation in BTBR, D3 + vehicle and D3 + shEfnA4 group for 4 days, the neurites labeled by phalloidin (green). (G) Quantification of area and the length of neurites in the three groups. D3 + shEfnA4 reduced the area and length of neurites compared to D3 + vehicle group. *n* = 40. (H) Western blots show for p‐PI3K, PI3K, p‐AKT and AKT on hippocampus lysates from the four groups (left). Quantification of these protein expression levels (right). β‐ACTIN was used as a loading control. *n* = 6. Data are presented as mean ± SEM.

To determine whether calcitriol‐mediated upregulation of the axon guidance factor EfnA4 is crucial for improving ASD, we assessed the effects of calcitriol on axon development through neural progenitor cell (NPC) migration and neurite outgrowth. We first measured the area covered by migrating NPCs. We found that BTBR NPCs exhibited decreased migration (~30%–50%) compared to C57 NPCs. As expected, calcitriol‐treated NPCs increased migration area compared to BTBR NPCs, which was abolished by EfnA4 knockdown in NPCs (Figure 4A–C, Figure S4A,B). Additionally, the difference in migration area was independent of the initial size of the neurospheres, indicating that calcitriol significantly promotes NPC migration in an EfnA4‐dependent manner (Figure 4D, Figure S4C). To further evaluate whether the absence of EfnA4 expression inhibits axon growth improvements induced by calcitriol, we induced neuronal differentiation of neurospheres on a homogeneous Poly‐D‐lysine (PDL) matrix. After 4 days, the longest neurites projecting from each sphere were measured and compared. We found calcitriol‐treated neurites growed significantly longer than BTBR neurites, while EfnA4 knockdown inhibited the growth‐promoting effects of calcitriol on neurites (Figure 4E–G). These findings suggested both impaired neurite outgrowth and migration in BTBR NPCs could be improved via targeting EfnA4 with calcitriol. Furthermore, Western blot analysis confirmed that calcitriol activates phosphorylation of the PI3K‐AKT pathway in BTBR‐derived NPCs, aligning with our in vivo results. However, EfnA4 knockdown inhibited this activation, suggesting that EfnA4 inhibition prevents the increase in PI3K‐AKT pathway phosphorylation in BTBR‐derived NPCs (Figure 4H, Figure S4D). Collectively, these results suggest that calcitriol rescues impaired neuronal growth and migration associated with BTBR‐ASD through activation of the PI3K‐AKT pathway via EfnA4.

### 3.5 Calcitriol Enhances Axon Guidance Cues via EfnA4‐EphA4 Reverse Signaling Pathway

**FIGURE 5:**
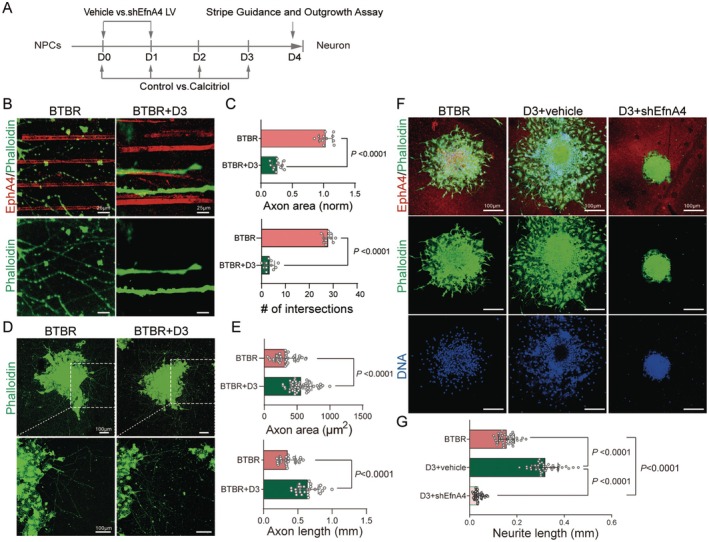
Calcitriol rescues axonal guidance defects in BTBR mice by promoting EfnA4‐mediated EphA4 signaling. (A) Timeline of the experimental procedures. (B, C) BTBR and BTBR + D3 neurospheres were cultured for 4 days on stripes of Fc‐tagged EphA4‐Fc and PDL. High magnification images and analysis of the thresholded area occupied by axons (Phalloidin, green) normalized to the area of axons growing in the BTBR group (norm), and axon crossings on EphA4‐Fc (anti‐Fc, red) stripes. *n* = 12. (D, E) BTBR and BTBR + D3 neurospheres were cultured for 4 days on PDL. Images and analysis of area and the length of axons in the two groups. BTBR + D3 axons appeared longer than those of BTBR axons. *n* = 40. (F, G) BTBR, D3 + vehicle and D3 + shEfnA4 neurospheres were cultured for 4 days on EphA4‐Fc (anti‐Fc, red) and PDL. Images and analysis of neurite length outgrowth in the three groups upon EphA4‐Fc cluster. D3 + shEfnA4 decreased the length of neurite growth compared to D3 + vehicle group. *n* = 40. Statistical significance is denoted as ****p < 0.0001. Data are presented as mean ± SEM.

Calcitriol effectively rescues the reduction in neurite numbers and the abnormal axon distribution observed in the hippocampus of BTBR mice. To elucidate the underlying mechanism, we investigated whether this effect is mediated by the EphA4 receptor through the EfnA4 reverse signaling pathway, which regulates axon behavior. We cultured neurospheres derived from the BTBR mouse hippocampus on alternating EphA4‐Fc (EfnA4 receptor) stripes while administering calcitriol treatment. After 4 days of directed neuronal differentiation, neurons were fixed and immunolabeled for Fc to visualize EphA4, as well as phalloidin to label F‐actin (Figure 5A). The results showed that BTBR neurons crossed EPhA4 stripes, while calcitriol‐treated BTBR axons are guided along with stripes of EphA4. It was clear that calcitriol‐treated BTBR neurons show little of the thresholded area occupied by axons and the axon crossings upon EphA4‐Fc stripes compared with BTBR neurons (Figure 5B,C). The BTBR neurons had short axons while calcitriol‐treated BTBR axons are longer. Meanwhile, the total area and length of axons extending were increased in calcitriol‐treated BTBR vs. BTBR (Figure 5D,E). Indeed, we observed that BTBR neurons showed no preference for EphA4‐Fc, confirming that EfnA4 was responsible for neurite guiding, rather than discontinuous EphA4‐Fc conjugate. Importantly, the knockdown of EfnA4 completely abolished the axon growth induced by calcitriol (Figure 5F,G). These findings suggest that calcitriol plays a critical role in maintaining axon guidance and synaptic activity through activation of the downstream PI3K signaling pathway via EfnA4.

### 3.6 Inhibition of EfnA4 Diminishes Calcitriol's Therapeutic Effects on ASD‐Like Behaviors

**FIGURE 6:**
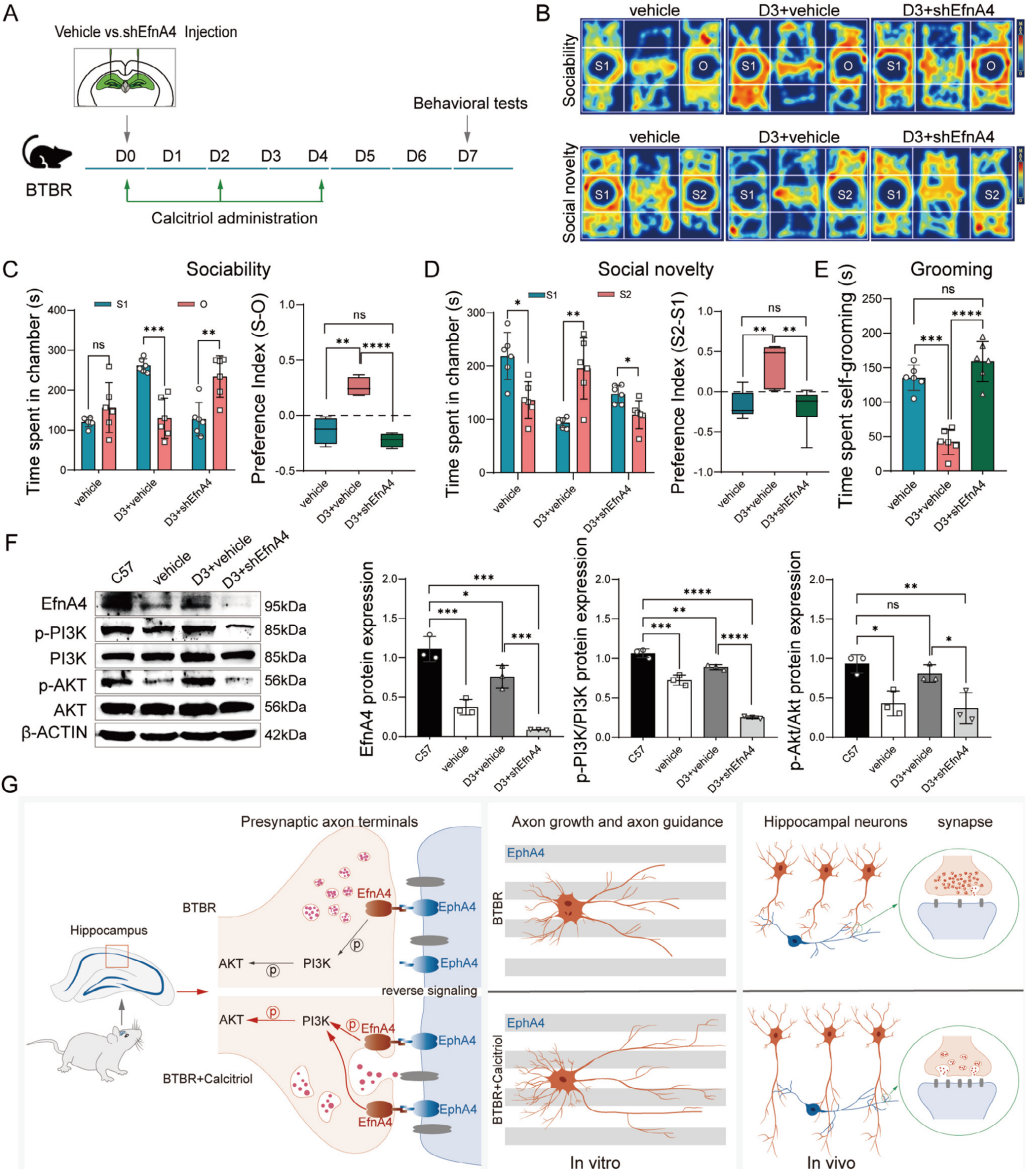
EfnA4 knockdown block the behavioral improvements induced by calcitriol treatment in BTBR mice. (A) Timeline of the stereotaxic surgery and autism‐related behavioral tests. (B) Representative tracing heatmap analysis of BTBR mice treated with vehicle (vehicle), calcitriol (D3 + vehicle) or combined calcitriol and shEfnA4 (D3 + shEfnA4) during the three‐chamber test. (C) Interaction time of the experimental subject with a stranger mouse or object in the sociability task (left). Social preference index revealed decreased social interaction in D3 + shEfnA4 mice compared with the D3 + vehicle group (right). *n* = 6. (D) Interaction time of the experimental subject with a familiar mouse (S1) or a novel mouse (S2) in the social novelty task (left). Social novelty index indicated that D3 + shEfnA4 mice spent less time with S2 than with S1 (right). *n* = 6. (E) Time spent on self‐grooming in the three groups. *n* = 6. (F) Western blots showed EfnA4, p‐PI3K, PI3K, p‐AKT, and AKT on hippocampus lysates from C57 mice, BTBR mice treated with vehicle (BTBR + vehicle), calcitriol (D3 + vehicle), and combined D3 and shEfnA4 (D3 + shEfnA4) (left). Quantification of these protein expression levels (right). Both the phosphorylation levels of PI3K and AKT were significantly increased in the D3 + vehicle group, while they were decreased in the D3 + shEfnA4 group. *n* = 3. (G) Models of calcitriol function in improving abnormal hippocampus axons in BTBR. The deficiency of EfnA4 in the hippocampus of BTBR mice reduced the sensitivity of axon terminals to EphA4 receptors, resulting in weakened EfnA4 retrograde signaling and decreased phosphorylation of PI3K/AKT, thereby causing abnormal axon elongation, guidance, and synaptic activity. Calcitriol promoted normal axon growth and synaptic activity by upregulating EfnA4 to enhance EfnA4‐EphA4 retrograde signaling and activating PI3K/AKT. Statistical significance is denoted as ns (no significance), *p < 0.05, **p < 0.01, ***p < 0.001, ****p < 0.0001. Data are presented as mean ± SEM.

To further investigate the role of calcitriol in promoting neurite growth and guidance in the hippocampus via EfnA4 in vivo, we administered shEfnA4 lentiviral vectors directly into the hippocampus of calcitriol‐treated BTBR mice (Figure 6A). The three‐chamber social test was utilized to evaluate the impact of EfnA4 deficiency on the social interactions of calcitriol‐treated BTBR mice. In the social interaction phase, shEfnA4‐treated mice spent significantly less time in the chamber with a novel mouse compared to the time spent in the chamber with a non‐social object, unlike the calcitriol‐treated BTBR mice (Figure 6B,C). During the social novelty test, shEfnA4‐treated mice displayed a preference for the first familiar novel mouse, spending more time in that chamber (Figure 6B,D). Furthermore, mice lacking EfnA4 exhibited excessive grooming behaviors (Figure 6E). These findings indicate that EfnA4 knockdown negates the beneficial effects of calcitriol on social interactions and repetitive behaviors in BTBR mice. Moreover, in vivo injection of shEfnA4 to inhibit EfnA4 effectively reversed the calcitriol‐induced activation of the PI3K‐AKT signaling pathway in the hippocampus (Figure 6F). Although the increase in AKT activation following calcitriol treatment was suppressed in the shEfnA4 group, shEfnA4 treatment did not affect the phosphorylation levels of ERK or PKC, which were also essential for EfnA4‐mediated reverse signaling (Figure S5A,B). This observation suggested that EfnA4 inhibition effectively prevents the enhanced phosphorylation of the PI3K‐AKT pathway in BTBR mice.

## 4 Discussion

This study investigates the potential therapeutic effects of calcitriol, the active form of VitD, in alleviating ASD‐like behaviors in BTBR mice, with a focus on underlying molecular mechanisms. Our findings provide compelling evidence that calcitriol treatment significantly improves core ASD‐related behaviors, hippocampal morphology, and synaptic activity in BTBR mice, underscoring its potential as a therapeutic agent for ASD. Notably, these effects appear to be mediated through activation of the EfnA4‐EphA4 reverse signaling pathway, which modulates axon guidance via the PI3K‐AKT pathway (Figure 6G).

VitD is critically involved in brain development and synaptic plasticity [22]. Previous studies have shown that maternal VitD deficiency can cause long‐lasting alterations in both the functional and anatomical structures of the brain in offspring [23, 24]. Furthermore, prenatal VitD deficiency has been associated with impairments in long‐term potentiation, synaptic plasticity, and learning and memory functions [25, 26]. These findings suggest that VitD influences neurodevelopment in key brain regions and plays an essential role in maintaining normal cognitive function through regulation of specific developmental factors [27]. In this study, we observed that calcitriol treatment significantly alleviated ASD‐related behaviors, improving sociability and social novelty preference in BTBR mice. These results are consistent with previous reports that calcitriol enhances social behaviors in various rodent models [28, 29]. Additionally, calcitriol reduced repetitive behaviors, such as excessive grooming and marble burying, further supporting its therapeutic potential in ameliorating core ASD symptoms. Notably, calcitriol supplementation rescued hippocampal hypoplasia, restored brain volume, and promoted dendritic growth across all examined regions. These morphological deficits were associated with impaired synaptic activity, as evidenced by ultrastructural analysis, which revealed reduced synaptic active zone length, postsynaptic membrane thickness, and synaptic cleft width in BTBR mice. Calcitriol treatment effectively reversed these morphological and synaptic abnormalities, suggesting that calcitriol not only promotes neurite growth but also enhances synaptic plasticity. These findings support the idea that VitD positively influences neuronal connectivity and social communication [30]. Our results align with studies showing that VitD supplementation ameliorates hippocampal deficits in various neurological disorders [31, 32].

To explore the molecular mechanisms underlying calcitriol's therapeutic effects, we performed transcriptomic analysis to identify differentially expressed genes in the hippocampus of calcitriol‐treated BTBR mice. We found a significant upregulation of genes involved in axon guidance, neuron migration, and central nervous system differentiation, consistent with calcitriol's established role in promoting neurogenesis and synaptic plasticity [33, 34]. Notably, the expression of EfnA4, an axon guidance factor, was reduced in the hippocampus of BTBR mice. However, calcitriol treatment upregulated EfnA4 expression and activated the PI3K‐AKT signaling pathway. While prior studies have identified abnormalities in axon guidance, neurogenesis, and actin cytoskeleton regulation in BTBR mice [35, 36, 37], few have specifically targeted axon guidance molecules. The identification of EfnA4 as a key upregulated gene in calcitriol‐treated mice further supports the involvement of the PI3K‐AKT pathway in mediating calcitriol's effects. We demonstrated that when calcitriol was added to BTBR hippocampus‐derived NPC cultures, EfnA4 expression was restored, leading to significant improvements in NPC migration and axonal growth. Silencing EfnA4 expression, however, reversed these beneficial effects, supporting our hypothesis that EfnA4 is a crucial mediator of calcitriol's impact on axon guidance and neurodevelopment. Meanwhile, our results also confirmed that the restoration of EfnA4 expression by calcitriol is a key factor in correcting both behavioral and anatomical deficits in BTBR mice, emphasizing the importance of this axon guidance molecule in ASD pathology.

Moreover, our results highlight the pivotal role of the PI3K‐AKT pathway in mediating EfnA4's effects on axon growth and guidance. It was reported that EfnA4 is a pleiotropic ligand that can interact with EphA receptors [38] and most Eph B receptors [39]. Ephrin‐Eph bidirectional signaling to regulates the interaction between migrating axons and surrounding guidance cues [40]. However, “reverse” signaling in ephrin‐expressing cells has been explored less than Eph receptor forward signaling. In our study, we found that EfnA4 promoted neurite outgrowth and axon guidance wiring through the negative regulation of EphA4 and upregulated the PI3K/AKT signaling pathway, but EfnA4 deficiency had no significant regulatory effect on the phosphorylation level of ERK. These results are in line with previous reports of AKT‐phosphorylation enhancement by EfnA4 reverse signaling in BDNF‐promoted retinal axon branching [41]. In addition, reverse signaling within EfnA4‐positive fibers in the ipsilateral cortex was reported to be mediated by a combination of receptors, including TrkB and EphA4 [42]. EfnA4‐dependent TrkB activation contributes to the AKT‐phosphorylation in CPA growth cones up to the threshold to assist axon growth. However, EfnA4 is also reported to interact with p75NTR and activated p75NTR to mediate axon repulsion [16, 43]. These studies suggest that EfnA4 reverse signaling elicits either cell repulsion or adhesion depending on context‐cellular pathways [44]. Different types of Ephrins or subtle changes in relative levels of Eph‐Ephrins can have marked effects on neuronal function and individual behavior [45]. Thus, our study further extends the role of EfnA4 in axon guidance by identifying the PI3K‐AKT pathway as a critical regulator of its effects on axon growth.

In conclusion, calcitriol enhances EfnA4 expression, activating the PI3K‐AKT pathway to promote neurite growth and migration, which is crucial for ameliorating ASD‐related phenotypes in BTBR mice. Given that the BTBR model is proposed to mimic the human behavioral impairments of autism [46, 47], these findings have potential implications for developing effective treatments for autism. Specifically, by identifying the EfnA4‐AKT signaling axis as a potential therapeutic target, this study provides new insights into the neurobiological mechanisms of ASD and the clinical application of VitD as a treatment strategy. Future studies will focus on investigating the use of calcitriol in early neurodevelopmental interventions to establish the optimal therapeutic window for ASD treatment.

## Author Contributions

Zhiyan Shan and Lei Lei conceived the project. Tiantian Gong, Chenyao He, and Xin Liu performed the experiments. Qi Jiang and Qi Wang helped to perform the molecular experiments. Yubo Qi, Jieli Bai, and Wenxin Ding helped to perform the immunofluorescence experiments. Tiantian Gong and Jingling Shen wrote the manuscript. Tiantian Gong and Ruizhen Sun made illustrations and generated figures. All other authors provided feedback on the manuscript. All author(s) read and approved the final manuscript.

## Ethics Statement

All experimental protocols were approved by Harbin Medical University and the Guangzhou National Laboratory Animal Committee (GZLAB‐AUCP‐2024‐08‐A03). All experimental protocols were conducted in accordance with the principles of laboratory animal care and the committee's guidelines.

## Conflicts of Interest

The authors declare no conflicts of interest.

## Supporting information


Appendix S1.

## Data Availability

The datasets are available from the corresponding author upon reasonable request.
