# Supplementary material for: Calcitriol Modulates Hippocampal Axon Guidance Through Enhanced EfnA4‐Mediated PI3K/AKT Signaling in an Autism Mouse Model
Source: CNS Neurosci Ther. 2025 May 21;31(5):e70429. doi: 10.1111/cns.70429 (PMC12093051; doi:10.1111/cns.70429)
Supplement: Supplementary file 1 — Appendix S1. [file CNS-31-e70429-s001.doc]

**Calcitriol Modulates Hippocampal Axon Guidance through Enhanced EfnA4-Mediated PI3K/AKT Signaling in an Autism Mouse Model**

**Supplemental information**

1. **Supplemental Materials and Methods**

**Three-chamber social test**

Mice were first habituated for 10 min in an empty (40 cm × 60 cm × 22 cm) transparent polycarbonate devices measuring divided into three interconnected chambers (left, center, and right). Sociability was evaluated during a second 10min period in which the subject could interact either with a wire cup containing an object (O) or a wire cup containing a stranger mouse (S1, same age and no previous contact with the subject). TM-Vision was used to track the total time in each chamber and sniffing time. Preference for social novelty was assayed in a third 10 min period by introducing a novel stranger mouse (S2) into the previously wire cup containing object. The time spent interacting with the familiar mouse or the novel mouse was recorded and measured using TM-Vision. Sociability index = Time (S1 − O) / (S1 + O), social novelty index = Time (S2 − S1) / (S2 + S1).

**Marble burying test**

The standard experimental cage (measuring 46.5 cm × 30 cm × 18.5cm) was filled with bedding material to a 5-6 cm depth. The squirrel cage was then gently moved to create a level surface. Next, twenty black glass marbles, each with a diameter of 1.5 cm, were carefully placed onto the cushion, forming a uniform 4×5 grid pattern. All mice were gently placed in the same corner of the cage and allowed to explore freely. After 30 min, the mice were removed from the cage and each cage was photographed for subsequent analysis. Any marbles with over 67% of their volume buried were recorded.

**Self-grooming test**

Self-grooming behavior was defined as stroking or scratching of the body or face, or licking body parts. Mice were individually placed in the cage. A clean cage without bedding was used and the experimental process included a 10 min habituation stage and a 10 min test stage. During the test stage, the cumulative self-grooming time was recorded using a stopwatch.

**Nissl staining**

Mice were perfused transcardially with saline, followed by 4% paraformaldehyde (PFA, Bodi Chemical). Then, the brains were harvested for fixation for 48-72h and were processed in the coronal plane with paraffin, and sectioned at 5μm. The brain sections were dewaxed and dehydrated with xylene, a graded series of alcohol, distilled water, and were treated with a nissl staining solution (Beyotime) for 10 min, washed, dehydrated again, and mounted with neutral balsam.

**Primary NPCs culture**

Primary NPCs were obtained from the hippocampi on the 7th [postnatal](../../../../C:%5CUsers%5C甜甜%5Cbaidu-translate-client%5Cresources%5Capp.asar%5Capp.html) C57 and BTBR mice. Briefly, the isolated tissues were minced in PBS, pipetted into the suspension and incubated in 0.05% trypsin-EDTA (Sigma-Aldrich) at 37 °C for 10 min for a single cell suspension. Add the trypsin inhibitor (Thermo Fisher Scientific) to stop the trypsin activity and plate cells in proliferation medium, DMEM/F12 (Thermo Fisher Scientific) supplemented with 20 ng/ml epidermal growth factor (EGF, PeproTech), 1% L-glutamine (Thermo Fisher Scientific), 1×B27 (Thermo Fisher Scientific), and 1% penicillin and streptomycin (Thermo Fisher Scientific). The neurospheres appeared after three days culture at 37°C in a humidified incubator with 5% CO_2_. After seven days, the spheres with 150–200 µm in diameter and were harvested for the following experiments. For adherent cultures, cells were cultured on Poly-L-Ornithine (PLO, Sigma, 15 µg/ml) and fibronectin (FN, Crgen, 10 µg/ml) coated dishes.

**Neural differentiation and culture**

Neurospheres were adherent cultures on acid-washed coverslips coated with Poly-D-lysine (PDL, Beyotime, 100 µg/ml). Following the first day, the whole media were changed to neural induction medium consisting of DMEM/F12 and neurobasal medium supplemented with N2 and B27. The neural induction medium was changed every two days.

**Neurosphere migration assay**

[Neurospheres](../../../../C:%5CUsers%5C甜甜%5Cbaidu-translate-client%5Cresources%5Capp.asar%5Capp.html) were cultured for 48h onto slides coated with PLO (15 µg/ml) and FN (100 µg/ml) to exhibit a contiguous migrating cell carpet and were imaged. These neurospheres were to measure migration in each group (C57, BTBR and calcitriol), and was repeated at least three experiments. Average migration area was quantified by subtracting initial sphere size (ISS) from the total area, and migration rate (%) was statistics by migration area to the total area using Image J.

Given that neurospheres were not the same size when plated, it was important to ensure that the ISS did not affect migration. Hence, a linear regression analysis was conducted to assess the correlation between neurosphere size and migration rate. The strength of the linear relationship between the variables was indicated by the correlation coefficient (R²), where values closer to ±1 signify a stronger positive or negative correlation.

**Neurite outgrowth assay**

[Neurospheres](../../../../C:%5CUsers%5C甜甜%5Cbaidu-translate-client%5Cresources%5Capp.asar%5Capp.html) followed culture onto slides coated with 100 µg/ml PDL and neural differentiation were plated for four days. Then, the cells were fixed in 4% PFA and stained by phalloidin. Neurites were defined as processes that extend from the cell body and are equal to or greater than two cell body diameters in length. Measurements of neurite lengths were made by tracing from the end of the neurite to the edge using the Image J software.

**Stripe guidance assay**

To evaluate axonal guidance cues, the stripe molds designed by our laboratory and produced by Guangdong Yanjia Laser Co., Ltd. Acid-washed coverslips were coated in PDL (100 µg/ml) for 3 h and rinsed extensively with DPBS before drying. Stripe mold was affixed and 50 µl of EphA4-Fc (10 µg/ml, MCE) fed through the mold and allowed to incubate for 1 h at RT. After extensive rinsing with PBS, molds were removed and the patterns were rinsed further with PBS. EphA4-Fc binding was independently confirmed with antibodies to Fc (AffiniPure Goat Anti-Human IgG, Fcγ Fragment Specific, Jackson). Neurons on patterned cues were labeled phalloidin.

**Lentiviral vector of shEfnA4**

The lentiviral vector expressing short hairpin RNA (shRNA) targeting the sequence of EfnA4 gene were purchased from SuZhou GENEWIZ Co., Ltd (Supplementary Table 2). Correct insertions of shRNA cassettes were confirmed by restriction mapping and direct DNA sequencing. The shRNA-expressing lentiviral was transfected into 293T cells together with the lentiviral packaging helper plasmids to generate respective lentiviruses. The lentivirus stock solution was collected 48 h post-transfection, then filtered through 0.45 µm filter. The ability of the four shEfnA4 vectors to knockdown EfnA4 was investigated using qPCR.

**2. Supplementary Figures**


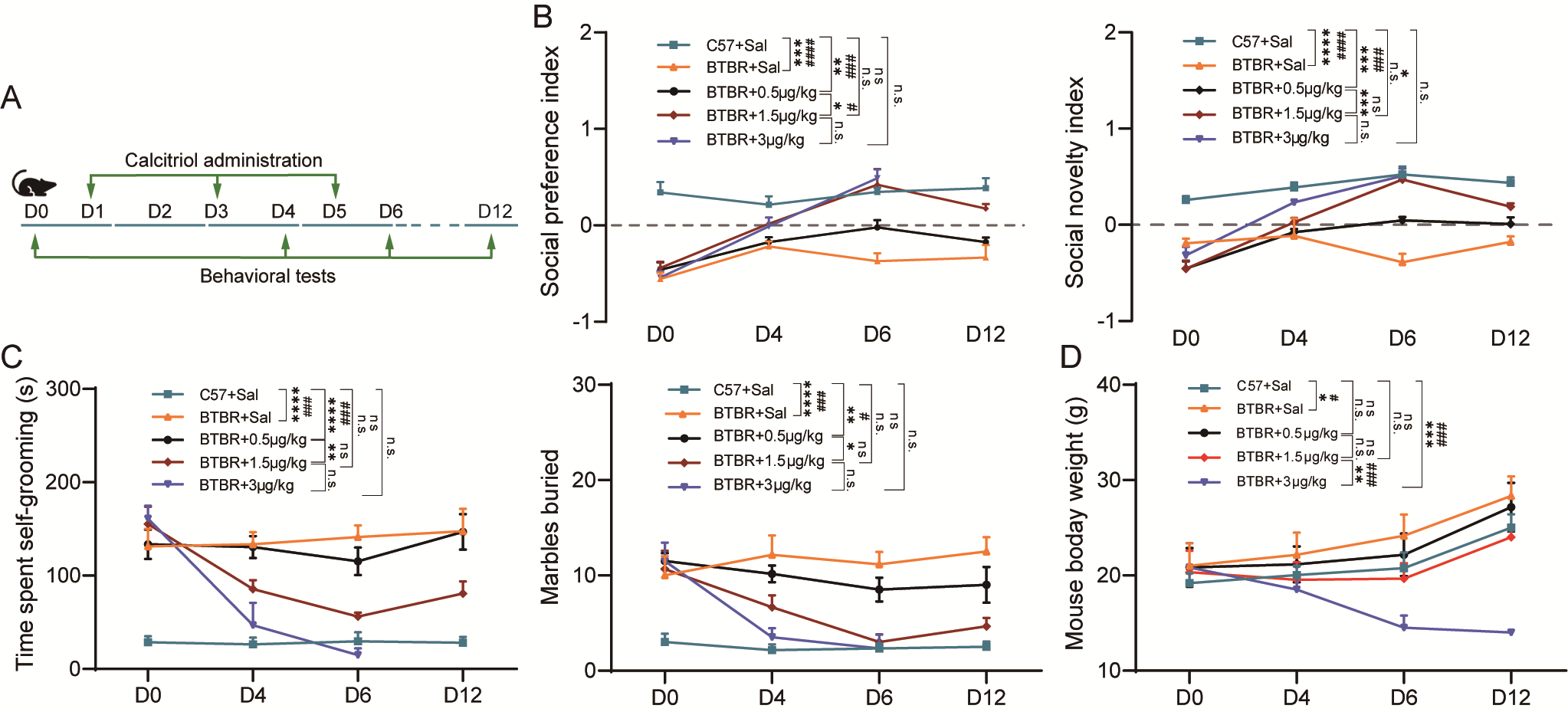
**S****upplemental Figure 1. The dose and time-dependent efficacy of calcitriol on BTBR behaviors.** **A** The schematic diagram shows the process of treating BTBR mice with calcitriol. Different doses of calcitriol (0.5μg/kg, 1.5μg/kg, 3μg/kg) were intraperitoneally injected into BTBR mice every other day for a total of three times. The normal saline injection groups of C57 and BTBR mice were used for comparative analysis. **B** Social preference index in stage of sociability (left) and social novelty (right) in the five groups. It showed that 0.5 μg/kg calcitriol treatment had no significant effect, while 1.5 μg/kg and 3 μg/kg could effectively improve the social behaviors of BTBR mice. **C** Grooming and marble burying test for the five groups. It showed that 0.5 μg/kg calcitriol treatment had no significant effect, while 1.5 μg/kg and 3 μg/kg could effectively improve stereotyped behaviors of BTBR mice. **D** Comparison of body weight among five groups of mice. It showed 3 μg/kg treatment group led to a sharp decrease in the body weight of most mice and even the death of some mice, while treatment with 0.5 μg/kg and 1.5 μg/kg calcitriol led to weight loss in mice, but did not affect survival. Statistical significance is denoted as n.s. or ns (no significance), *^/#^*P* < 0.05, **^/##^*P* < 0.01, ***^/###^*P* < 0.001, ****^/####^*P* < 0.0001. n.s. and **P* indicate analysis at D6*,* ns and ^#^*P* indicate statistical analysis at D12. Data are indicated as mean±SEM (n = 6).


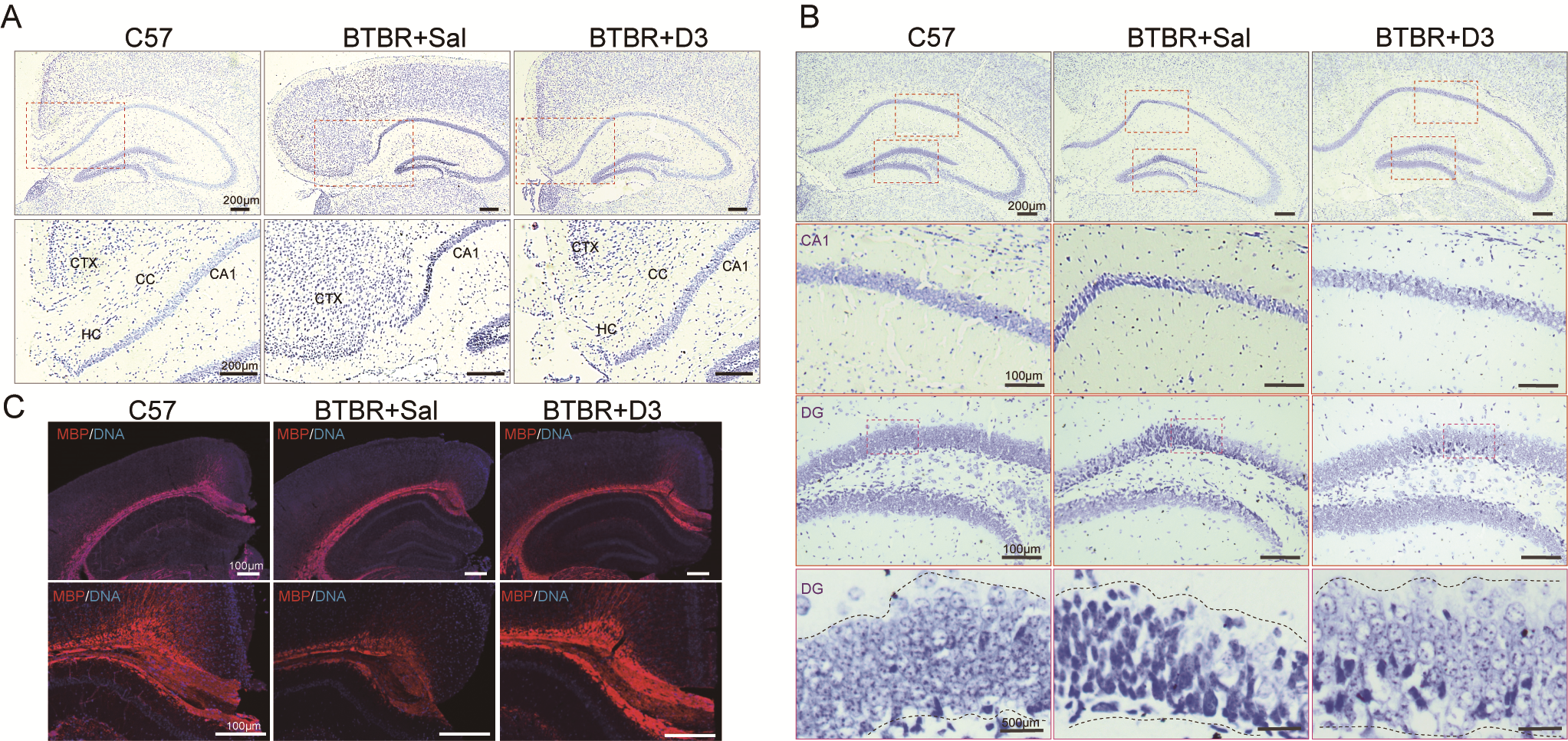


**Supplemental Figure 2.** **Calcitriol improves neuroanatomical differences between BTBR and C57 mice. A** Nissl staining was conducted on the coronal sections of the brain from samples of C57, BTBR+Sal, and BTBR+D3. The hippocampus of BTBR presented distinct developmental deficiencies, encompassing abnormal cortex (CTX), minute corpus callosum (CC), and malformed hippocampal commissure (HC). In contrast to the BTBR group, the brains of BTBR+D3 demonstrated remarkable improvements in these neuroanatomical structures. **B** Nissl staining reveals the developmental conditions of different hippocampal regions in the three group. BTBR+D3 enhanced the thickness of the CA1 pyramidal layer and DG granule layer in BTBR hippocampus. **C** Immunostaining of MBP^+^ neurons in CC in the three groups.


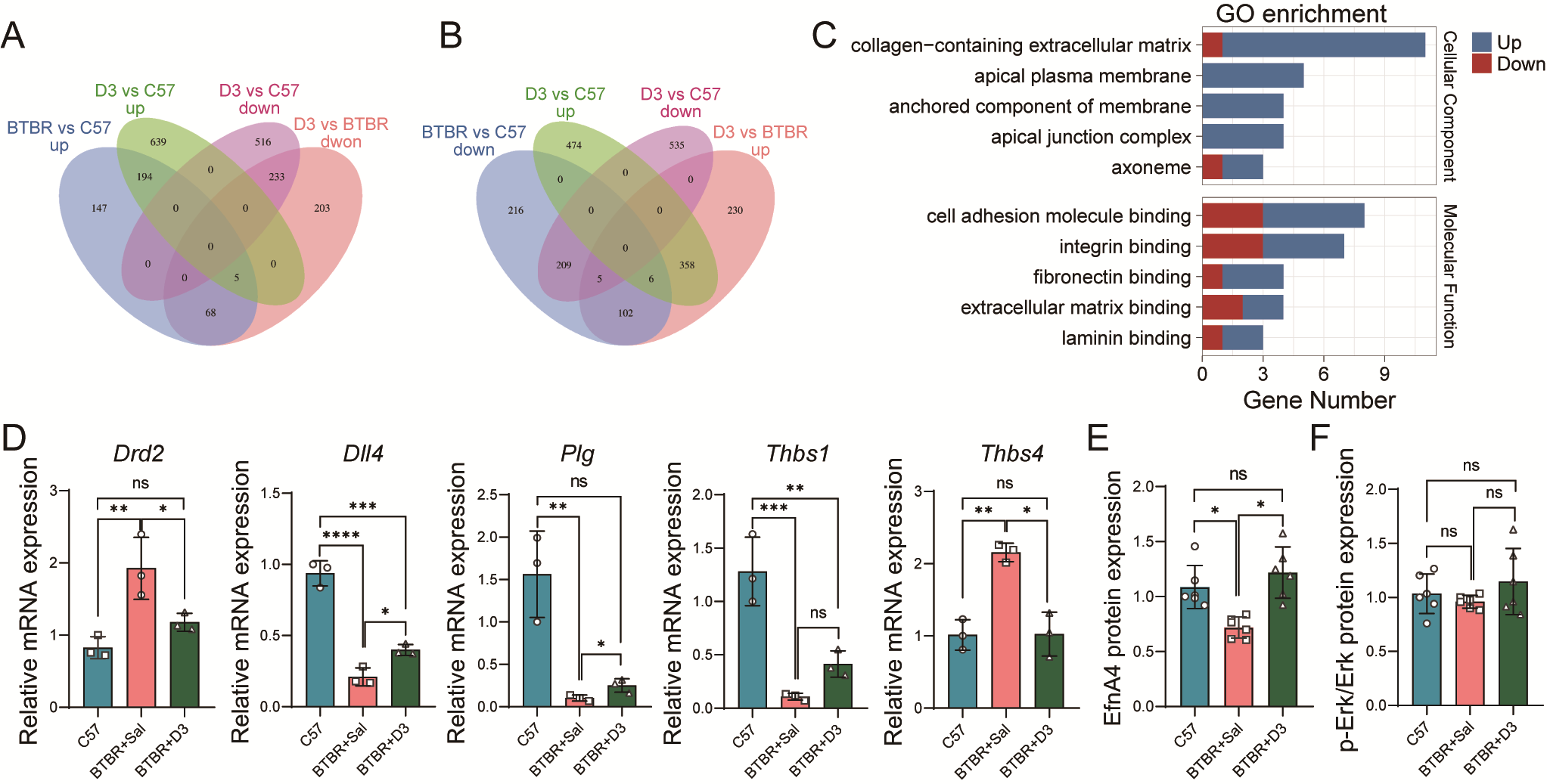


**Supplementary Figure 3. Calcitriol normalizes the expression of genes associated with axon guidance. A** Venn diagram illustrating the overlap among the up-regulated genes (BTBR vs C57, D3 vs C57) and down-regulated genes (D3 vs BTBR, D3 vs C57). **B** Venn diagram illustrating the overlap among the up-regulated genes (D3 vs C57, D3 vs BTBR) and down-regulated genes (BTBR vs C57, D3 vs C57). **C** GO analysis for terms indicating the cellular component and molecular function enriched in 170 differential expression genes in BTBR+D3 vs BTBR+Sal. Blue, up-regulated genes; red, down-regulated genes. **D** qPCR analysis shows the expression levels of key genes associated with cell migration (Drd2, Dll4), extracellular matrix (Plg) and integrin binding (Thbs1, Thbs4) signaling pathway in C57, BTBR+Sal and BTBR+D3 hippocampus, respectively. n = 3. **E** The protein expression of EfnA4 in C57, BTBR+Sal and BTBR+D3 hippocampus. **F** The phosphorylated expression levels of ERK in C57, BTBR+Sal and BTBR+D3 hippocampus. n = 6. Statistical significance is denoted as ns (no significance), **P <* 0.05, ***P <* 0.01, ****P <* 0.001, *****P <* 0.0001. Data are presented as mean ± SEM.


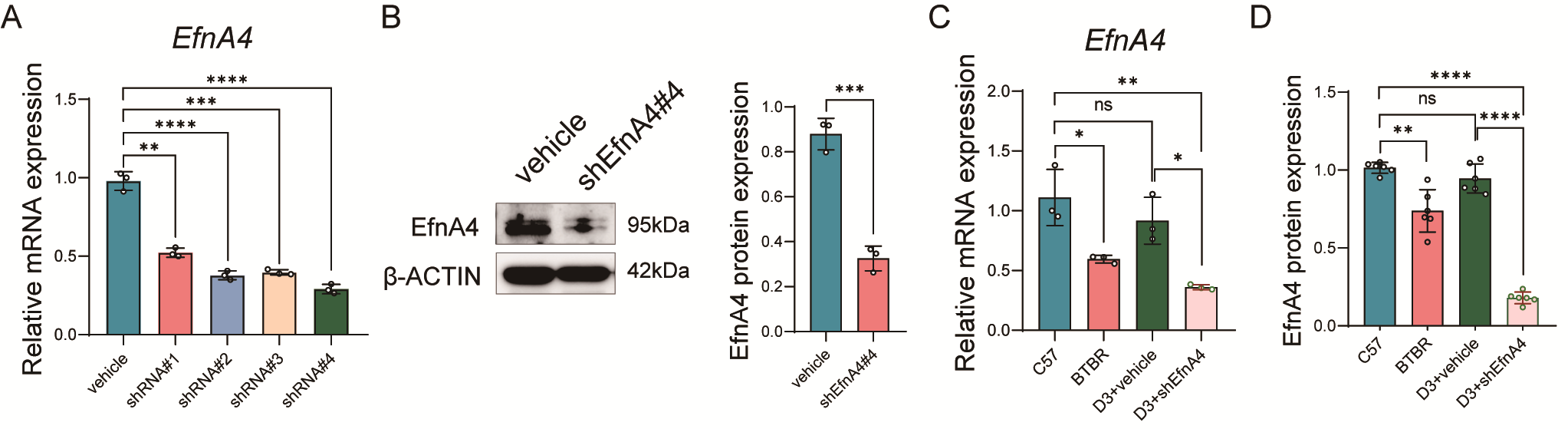


**Supplementary Figure 4.** **EfnA4 knockdown blocks the improvement of BTBR NPC migration and neurite outgrowth mediated by calcitriol. A** The mRNA expression of EfnA4 in NIH3T3 cells transfected with different shRNA virus. **B** The protein expression of EfnA4 in NIH3T3 cells transfected with different shRNA virus. n = 3. **C** The mRNA expression of EfnA4 in hippocampal NPCs from C57 mice, BTBR mice, BTBR mice treated with D3 and vehicle (D3+vehicle), BTBR mice treated with D3 and shEfnA4 (D3+shEfnA4) . **D** The protein expression of EfnA4 in the four groups. n = 6. Statistical significance is denoted as ns (no significance), **P <* 0.05, ***P <* 0.01, ****P <* 0.001, *****P <* 0.0001. Data are presented as mean ± SEM.


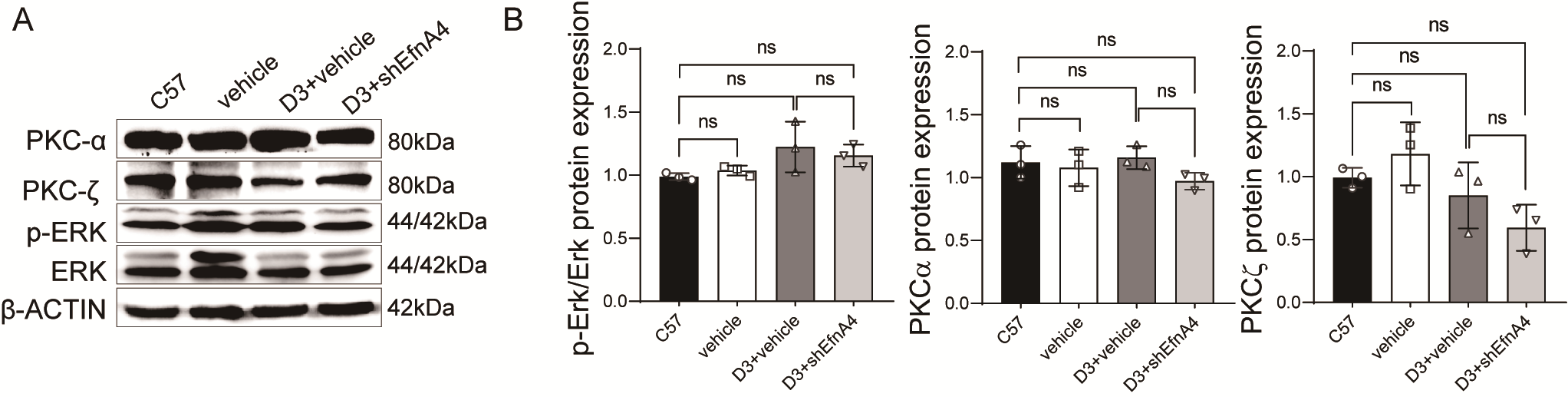


**Supplementary Figure 5.** **Calcitriol improves autism-like behaviors in BTBR mice via the EfnA4-PI3K/AKT signaling pathway.** **A** Western blots show for PKC-α, PKC-ζ, p-ERK, ERK protein on hippocampus lysates from C57 mice, BTBR mice treated with vehicle (BTBR+vehicle), BTBR mice treated with D3 and vehicle (D3+vehicle), BTBR mice treated with D3 and shEfnA4 (D3+shEfnA4). **B** Quantification of these protein expression levels reveals no significant difference in the four groups. n = 3. Statistical significance is denoted as ns (no significance), **P <* 0.05, ***P <* 0.01, ****P <* 0.001, *****P <* 0.0001. Data are presented as mean ± SEM.

**3. Key Resources Table**

**Supplementary Table 1. Antibodys**

| **Primary antibody** | **Company** | **Catalog number** | **Host species** | **Concentration** |
| --- | --- | --- | --- | --- |
| AffiniPure Goat Anti-Human IgG, Fcγ Fragment Specific (min X Bov,Hrs,Ms Sr Prot) | Jackson | 109-005-098 | Goat | 1：250 |
| EphrinA4 polyclonal Antibody | UpingBio | YP-Ab-15901 | Rabbit | 1：1000 |
| ERK1/2 mouse Monoclonal Antibody(1H4) | UpingBio | YP-Ab-14263 | Mouse | 1：1000 |
| Phospho-PI3 Kinase p85 (Tyr458)/p55 (Tyr199) Antibody | Cell Signaling | 4228T | Rabbit | 1：1000 |
| Phospho-Akt (Ser473) (D9E) XP® Rabbit mAb | Cell Signaling | #4060 | Rabbit | 1：1000 |
| Phospho-p44/42 MAPK (Erk1/2) (Thr202/Tyr204) Antibody | Cell Signaling | #9101 | Rabbit | 1：1000 |
| PKC Alpha Antibody | Cell Signaling | #2056 | Rabbit | 1：1000 |
| PKC Zeta Polyclonal antibody | Proteintech | 26899-1-AP | Rabbit | 1：1000 |
| PI3 Kinase p85 Alpha Monoclonal antibody | Proteintech | 60225-1-Ig | Mouse | 1：1000 |
| AKT Monoclonal antibody | Proteintech | 60203-2-Ig | Mouse | 1：1000 |
| Beta Actin Monoclonal antibody | Proteintech | 66009-1-lg | Mouse | 1：1000 |
| MAP2 Polyclonal Antibody | Thermo Fisher Scientific | PA1-10005 | Chicken | 1: 200 |
| Myelin Basic Protein (D8X4Q) XP® Rabbit mAb | Cell Signaling | #78896 | Rabbit | 1: 50 |
| **Secondary antibodies** | **Company** | **Catalog number** | **Host species** | **Concentration** |
| Goat Anti-Rabbit IgG H&L (HRP) | Abcam | Ab6721 | Goat | 1:2500 |
| Donkey Anti-Mouse IgG Antibody, HRP conjugate, Species Adsorbed | Merck | AP192P | Donkey | 1:2500 |
| Donkey anti-Chicken IgY-AF488 Antibody | Absin | abs20019 | Donkey | 1:100 |
| Goat-Anti-Rabbit H&L (TRITC) | GSZB-BIO | ZF-0316 | Goat | 1:50 |
| Rabbit-Anti-Goat IgG H&L (TRITC) | GSZB-BIO | ZF-0317 | Rabbit | 1:50 |

**Supplementary Table2. QPCR primers and shRNA sequence**

| **Primers** | **Forward** | **Reverse** |
| --- | --- | --- |
| EfnA4 | CCTGGAGAAAGCGGTACGTC | TCAGGAGACGGAGGATTGGG |
| Lmx1a | AATGGTAGTGGGAATGCGGG | TGGTCAATGGGGTTTCCCAC |
| Ntn5 | ATGTCTCCGTCAGCAGTGTG | CCTGGGCGTGCAGAACATAA |
| Lamc2 | CAGACACGGGAGATTGCTACT | CCACGTTCCCCAAAGGGAT |
| Drd2 | CCATTGTCTGGGTCCTGTCC | GCAGCATCCTTGAGTGGTGT |
| Dll4 | CCAGCAACCCCTGTCGAAAT | CAGGCATAACTGGACCCCTG |
| Plg | CAACCGCGTCGAGTATCTGA | GCCAAGACCCCAAGAAGTGA |
| Thbs1 | GGGGAGATAACGGTGTGTTTG | CGGGGATCAGGTTGGCATT |
| Thbs4 | TGGTGATCAGACGAGGGGAT | CACAGACATAGCCGTCACCA |
| sh-EfnA4#1 | CCGGGGAGCTGGGCTTCAACGATTACTCG  AGTAATCGTTGAAGCCCAGCTCCTTTTTG | AATTCAAAAAGGAGCTGGGCTTCAACGAT  TACTCGAGTAATCGTTGAAGCCCAGCTCC |
| sh-EfnA4#2 | CCGGGCCAAGAAGCAAAGGAGAAGCCTC  GAGGCTTCTCCTTTGCTTCTTGGCTTTTTG | AATTCAAAAAGCCAAGAAGCAAAGGAGAA  GCCTCGAGGCTTCTCCTTTGCTTCTTGGC |
| sh-EfnA4#3 | CCGGGCCCGGAAACCTTTGCATTAT CTCG  AGATAATGCAAAGGTTTCCGGGCTTTTTG | AATTCAAAAAGCCCGGAAACCTTTGCATTA  TCTCGAG ATAATGCAAAGGTTTCCGGGC |
| sh-EfnA4#4 | CCGGGGACCAAACCTTCAATAAATCCTCG  AGGATTTATTGAAGGTTTGGTCCTTTTTG | AATTCAAAAAGGAGCTGGGCTTCAACGAT  TACTCGAGGATTTATTGAAGGTTTGGTCC |
